# The effect of magnitude in a simultaneous duration assessment task among children – a replication study

**DOI:** 10.1371/journal.pone.0285564

**Published:** 2023-05-09

**Authors:** Amihai Gottlieb, Dan Zakay

**Affiliations:** The School of Psychological Sciences, Tel-Aviv University, Tel-Aviv, Israel; University of Melbourne, AUSTRALIA

## Abstract

The "magnitude effect" refers to the phenomenon where stimuli of greater magnitude appear to last longer in duration. Previous studies have explored this effect among children using various duration assessment tasks, but the findings have been inconsistent. Moreover, no replication studies have been conducted on this topic among children thus far. The simultaneous duration assessment task, which is one method for investigating time perception, has been used only twice in children and produced the magnitude effect. Thus, we aimed to replicate these findings and validate them through an additional replicated study. For these aims, we recruited 45 Arab-speaking children aged 7–12 to participate in two studies. In Study 1, they were asked to perform a simultaneous duration assessment task, where they had to assess the illumination durations of lightbulbs with strong and weak intensities simultaneously. In Study 2, they were asked to perform a duration reproduction task, where they had to reproduce the durations of illumination of the same stimuli. Both studies found a magnitude effect pattern, where the children tended to report that the lightbulb with the stronger intensity was illuminated for a longer duration or had a strong tendency to not choose the lightbulb with the weaker intensity. These results are discussed in terms of possible explanations for the conflicting results found in previous literature, as well as their consistency with the pacemaker model’s explanation for the effect.

## Introduction

The magnitude effect is one of the most robust and puzzling of all prospective-time related effects. Essentially, it describes where stimuli with more magnitude on a temporal-task unrelated dimension, such as perceived size, pitch, numerical value, symbolic size, etc., seem to appear for longer durations than stimuli with less magnitude on the temporal-task unrelated dimension, although their objective duration (clock time) is identical. While the magnitude effect has been extensively studied among adults [1], there have also been efforts to investigate its manifestation among children. Given that the effect pertains to a distortion of time perception, the magnitude effect among children is thought to be linked to developmental factors.

Among children, some experiments in the literature have added the ages of the children as an independent factor to the analysis. The results have been contradictory, with some studies finding that the magnitude effect is negatively correlated with age [2–6], while others report that it is independent of age [7, 8]. In one case, it was even found to be positively correlated with age [9]. These discrepancies could be due to the use of different durations, time assessment tasks, and analytical methods across the studies.

In some studies investigating the magnitude effect among children, language and cultural background were included as independent factors, while in others they were simply part of the participants’ demographic attributes. As a result, the effect was tested on children from various countries, including English, French, Italian, Hebrew, Greek, Dutch, and Portuguese speakers. Conflicting findings were also reported in these cases. For example, a group of researchers have found that Greek, Dutch, and Brazilian Portuguese speaking children aged 4–10 show a similar asymmetrical interference, where only magnitude affects time perception, while using language as an independent factor [7, 8]. Conversely, other researchers have reported that this asymmetrical relation decreases with age in a convenience sample of 5 to 8-year-old French speaking children [2]. Moreover, While most studies have demonstrated standard magnitude effects, a study conducted by Mioni and Stablum [6] on a convenience sample of Italian speaking children reported a reversed effect where larger magnitudes resulted in shorter perceived durations. These conflicting results could also be attributed to differences in methods and procedures used across studies.

Another factor that distinguishes studies of the magnitude effect among children is the duration assessment method used. Researchers such as Droit-Volet, Clément [2], Levin [4], Levin and Gilat [5], Droit-Volet and Coull [9], and Casasanto, Fotakopoulou [8] reported magnitude effects while using verbal responses e.g. "choose the stimuli that traveled for the longest duration" or by pressing a button for temporal bisection tasks, e.g., "press the button that corresponds with a longer interval". On the other hand, studies by Mioni, Stablum [6], Matsuda [10], Matsuda [11], and Zakay [12] used duration reproduction assessment tasks and still observed magnitude effects.

One rarely used duration assessment method in the context of magnitude effect exploration among children was employed by Levin [4] and Levin and Gilat [5]. This is the simultaneous duration assessment task. In this type of task, the participants are typically asked to assess the duration of two or more stimuli that were simultaneously presented for certain intervals and to choose which was presented longer (or shorter) (Levin [4], Levin and Gilat [5]), to reproduce these duration (e.g., Brown and West [13], Cheng, Yang [14], Hallez, Monier [15]) or to simultaneously produce two or more intervals (e.g., Brown and West [13], Klapproth [16]). As an example of such a task, participants might be asked to reproduce the durations of two figures presented in parallel for a few seconds, each for a different duration, a task that requires prospectively assessing the durations of both stimuli simultaneously. In their studies, Levin (1979) and Levin and Gilat (1983) investigated how time-related and unrelated cues affected the ability of children aged 4–7 to simultaneously compare durations. To do this, they tested the impact of stimuli speed or light intensity magnitudes [4] on the comparison, or the additive effect of two types of magnitudes [5]. The results showed that unrelated cues, such as the size or intensity of the lightbulbs, had a non-additive effect on perceived duration. In other words, larger magnitudes in time-irrelevant dimensions were treated as time-related cues and induced longer perceived durations. This effect was labeled "more is more" since larger magnitudes led to longer perceived durations. Thus, the researchers reported magnitude effects.

To our knowledge, there have been no replications of Levin’s studies, and on that matter, no other prior replication studies have been conducted in the context of the magnitude effect among school-age children between the ages of 6 and 12. Additionally, the simultaneous duration assessment task has only been used twice in this population, while other duration assessment methods have been used more frequently. Therefore, it is crucial to replicate these studies to confirm the findings and ensure that the results are robust and not due to chance or other factors. In order to do so, it is essential to verify the results using a widely accepted duration assessment method, such as a duration reproduction task. Moreover, replication studies are particularly important when studying children, as their responses may be influenced by various factors, including age, gender, and cultural background, given the conflicting findings related to language and age factors. Hence, replicating these studies in diverse cultural settings and among different age groups would enhance the external validity of the results obtained by Levin [4] and Levin and Gilat [5] and potentially help clarify some of the conflicting results observed.

### The current studies

The objective of this study is to replicate the findings of Levin [4] and Levin and Gilat [5] in diverse cultural settings and among different age groups. Additionally, the study aims to validate these findings using another duration assessment task, through replication of a study previously conducted by Zakay [12]. In Zakay’s study, 7–9 year old children were asked to estimate the lighting times of a small and low intensity (4 μW) and a large and strong intensity (12 μW) lightbulbs by reproducing their lighting times (3 and 6 seconds) with a flashlight.

For convenience reasons, we were able to conduct the replication studies among 45 7-12-year-old Arabic-speaking children living in a peripheral Arab village (Dir-Hana) in Israel. The studies were conducted by Ms. Judy Roan in her native village as part of her degree work at the Interdisciplinary Center, Herzliya.

## Study 1

### Materials and methods

#### Participants

45 children (aged 7–12) in the first, fourth, and sixth grades, 15 from each grade, were recruited for this study as a convenience sample. All of them spoke Arabic as their first language. Due to logistic and cultural reasons, ages and other demographics were not recorded. All children were males also due to logistic and cultural reasons. This study was approved by the Reichman University Ethics Committee. A written informed consent was obtained from all parents prior to their children participating in the study.

#### Material

Two light bulbs were used as the study stimuli: a high-intensity 100W and a low-intensity 40W. The intensities were selected as a convenience, taking into account the fact that for a magnitude effect to be demonstrated, only a relative difference in intensities is needed [17–20]. There were two duration conditions, 30 and 20 seconds. These durations were selected since they are longer than the "psychological present" [21–23], but remain in the range of what is commonly used in prospective duration studies [24].

#### Design and procedure

In this study, we partly adapted the procedure from Levin Levin [4] & Levin and Gilat [5] in which children were asked to judge the duration of two light at the same time.

All the participants were recruited while in school and received a verbal explanation about the study before taking part. Each participant was administered the task in a one-time one-on-one session with the experimenter. First, the experimenter provided a brief explanation, stating "In each trial, I will turn on two light bulbs for a certain duration. You will be asked to report if one lightbulb was turned on longer than the other and if so, which one, or if both were turned on for the same duration, according to your judgment". In each trial, the experimenter turned on the lightbulbs for a fixed duration and then turned them off. In all cases, the experimenter turned the light bulbs on and off at exactly the same time; i.e., for the exact same interval. The spatial location of the light bulbs, i.e., the relative position of the high and low bulbs, was random across participants. There were two duration conditions, 20 and 30 seconds, which were performed in two trials i.e., two trials per participant. On each trial, there were always two lightbulb intensities, 100W and 40W. The two duration conditions were conducted sequentially in this order: 20 seconds and then 30 seconds.

#### Statistical analysis

Chi-square analysis was used to examine the children’s overall decision frequencies for a potential general magnitude effect, assessing whether they tended to perceive the large intensity lightbulb as illuminated for a longer duration or avoided choosing the low intensity lightbulb. Additional chi-square analyses were conducted to examine the effects of duration, age, and their interaction on decision frequencies. Specifically, decision frequencies were analyzed at different levels of the duration and age factors, and the same effects were tested. All statistical analyses were performed using SPSS Statistics for Windows version 23 (SPSS Inc, 2008).

### Results

Table 1 shows the Distribution of the decisions for lit bulb durations as a function of the participants grades and the durations length conditions.

**Table 1:** Distribution of the decisions for lit bulb durations as a function of age and duration conditions.

Grade		Duration	Low Intensity < Large Intensity		Low Intensity > Large Intensity		Equal durations	
1^st^	Total	20 Seconds	15 (50%)	3 (20%)	11 (36.6%)	10 (66.7%)	4 (13.3%)	2 (13.3%)
		30 seconds		12 (80%)		1 (6.7%)		2 (13.3%)
4^th^	Total	20 Seconds	11 (36.6%)	4 (26.7%)	1 (3.3%)	1 (6.7%)	18 (60%)	10 (66.7%)
		30 seconds		7 (46.7%)		0 (0%)		8 (53.7%)
6^th^	Total	20 Seconds	8 (26.6%)	4 (26.7%)	1 (3.3%)	1 (6.7%)	21 (70%)	10 (66.7%)
		30 seconds		4 (26.7%)		0 (0%)		11 (73.3%)
Total	Total	20 Seconds	34 (37.7%)	11 (24.4%)	13 (14.4%)	12 (26.6%)	43 (47.7%)	22 (48.8%)
		30 seconds		23 (51.1%)		1 (2.2%)		21 (46.6%)

Regarding the presence of a general magnitude effect, we performed a Chi-square analysis on the distribution of total decisions, which yielded a significant result (Pearson’s chi-square (2) = 15.8, p < 0.001). The significant result was mainly driven by a strong tendency of the children to choose equal durations and an even stronger tendency to not choose Low Intensity>Large Intensity. This trend may be interpreted as a magnitude effect by negation.

To investigate the main effect of duration, we conducted the same analysis in both duration conditions. Only one significant result was observed in the 30-second duration condition (Pearson’s chi-square (2) = 19.7, p < 0.001). The significant result was mainly driven by a strong tendency of the children to not choose Low Intensity>Large Intensity and similar tendencies to choose equal and Low Intensity<Large Intensity. This trend may also be interpreted as a magnitude effect.

The analysis of the main effect of age, which examined the distribution of decisions across the three age groups, revealed a significant finding (Pearson’s chi-square (4) = 29.0, p < 0.001). Among the 4th and 6th graders, this result was consistent with the pattern of findings from the duration analysis. However, the 1st graders showed a tendency to choose both Low Intensity>Large Intensity and Low Intensity<Large Intensity, which suggests a different pattern of decision-making compared to the older age groups.

To explore the potential interaction between age and duration, the same analysis that was performed for age was repeated while dividing for the 20 and the 30 seconds conditions. These analyses yielded two significant results (Pearson’s chi-square (4) = 40.8, p < 0.001, Pearson’s chi-square (4) = 50.7, p < 0.001, respectively). These results were primarily driven by the behavior of first graders, who tended to choose Low Intensity>Large Intensity in the 20 seconds condition and Low Intensity<Large Intensity in the 30 seconds condition. This suggests a reversed magnitude effect in the former condition and a classic one in the latter.

### Discussion

The study results show a strong tendency not to choose Low Intensity over Large Intensity, suggesting the presence of magnitude effects. These patterns are consistent with the findings of Levin [4] & Levin and Gilat [5] where magnitude effects were demonstrated i.e., there was an association between the intensity of the light [4, 5] and the size of the lightbulb [5] with their duration, where larger magnitude stimuli were deemed to appear to last for longer durations.

The study revealed three noteworthy findings that are inconsistent with earlier research conducted by Levin [4] and Levin and Gilat [5]. First, the findings that a magnitude was not demonstrated in the shorter used duration. Second, the finding that the 1^st^ graders tend to decide that either one of the bulbs remained lit for a longer duration. Third, a reversed magnitude effect was obtained among the 1^st^ graders in the 20-seconds condition. All of these results that are inconsistent with the results of Levin [4] & Levin and Gilat [5], who reported on a magnitude effect which was more prominent among younger children and which dissipated with age. As Levin’s studies were conducted on children aged 4–7 and the current study was carried out on children aged 7–12, it is possible that the discrepancies between the current findings and Levin’s results are due to age-related developmental factors. The General Discussion section elaborates on these results in greater detail.

## Study 2

### Materials and methods

#### Participants

As in study 1.

#### Material

As in study 1, two light bulbs were used as the study stimuli: a high-intensity 100W and a low-intensity 40W. For the participants to reproduce the durations the bulbs stayed lit; each used a flashlight. To record the durations, the experimenter used a timer.

#### Design and procedure

In this study, we partly adapted the procedure from experiment 2 of Zakay [12] in which children were asked to reproduce the duration of two light bulbs with two different intensities and sizes.

First, the experimenter provided a brief explanation. She told the children that "in each trial, I will turn on one of the lightbulbs for a certain time and You will be asked to estimate for how long the bulb has been lit. Once I turn off the lightbulb, you will be asked to reproduce that duration by turning on the flashlight for that same amount of time". After the explanation, the participants were shown how to operate the flashlight, and it was made sure that they understood the reproduction method. In each trial, the experimenter turned on the lightbulb for a fixed duration and then turned it off. Then, the child was instructed to begin reproduction. Once he turned on the flashlight, the experimenter turned on a timer, and once the child turned the flashlight off, the timer was stopped. The experimenter recorded the duration that the timer measured and moved to the next trial. The children were asked to refrain from counting the seconds and instead attempt to internally assess the durations. As in study one, there were two duration conditions, 20 and 30 seconds, i.e., two trials per participant. The two durations, together with the two lightbulb intensities, 100W and 40W, created a 2X2 design, that was conducted sequentially in this order: 100W bulb for 30 seconds, 40W for 30 seconds, 100W for 20 seconds, 40W for 20 seconds.

#### Statistical analysis

In accordance with the Zakay [12] method of results analysis, the reproduced durations were subjected to a 2 (Durations: long/Short) × 2 (Bulb intensity: high/low) × 3 (Grade: First, fourth and sixth) mixed-design analysis of variance (ANOVA), with Duration and Bulb intensity as within-participant factors, and Grade as a between-participants factor. When violations of the assumption of sphericity were observed, Greenhouse-Geisser estimates were used to correct the degrees of freedom. Effect sizes were computed as partial eta squared and were evaluated based on Cohen’s benchmarks for interpretation [25]. All analyses were conducted using SPSS Statistics for Windows, version 23 (SPSS Inc, 2008).

### Results

**Fig 1:**
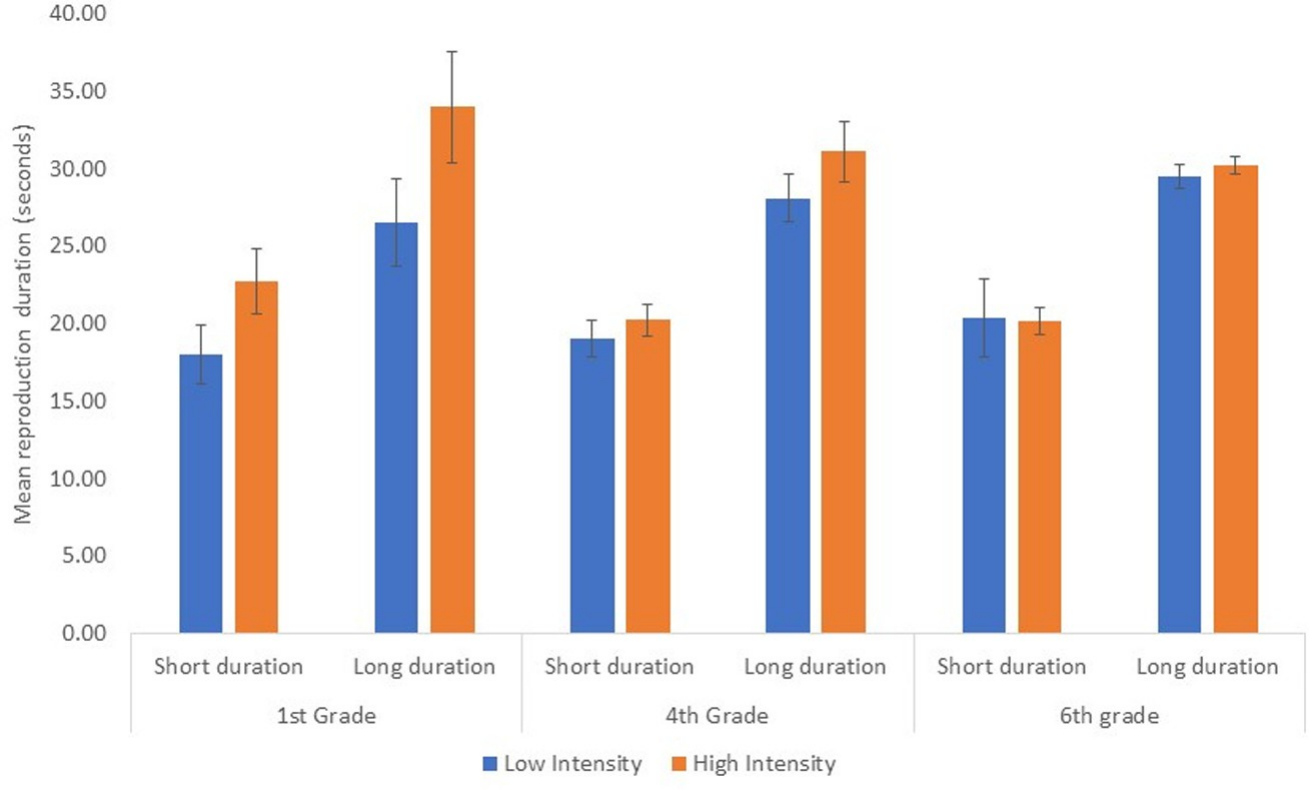
Durations reproduced by the children in each grade as a function of lit durations and bulb intensity. Error bars: 95% confidence interval.

Fig 1 displays the means and standard deviations of the durations reproduced by the children in each grade for the high and low intensities light bulbs and for the long and short durations.

As expected, the analysis revealed a significant large main effect for Duration, *F*(1, 42) = 1558.4, *p* < .001, ηp2 = .97. Pairwise comparisons indicated that there was a difference between the assessments of long and short durations (29.9 vs. 20.1 seconds, respectively, >.001). Critically, there was a significant large main effect for Intensity, *F*(1, 42) = 70.2, *p* < .001, ηp2 = .62, where the lit durations of the high intensity bulbs were judged to be longer than the lit durations of the low intensity bulbs (26.4 vs. 23.6, respectively, p < .001). Although there was no significant main effect of Grade, *F*(2, 42) = 2.4, *p* = .01, ηp2 = .10, there was a significant interaction between Intensity and Grade, *F*(2, 42) = 26.0, *p* < .001, ηp2 = .55, indicating that the younger the student, the greater the effect of the lightbulb’s intensity on the reproduced durations (a mean difference of 6 seconds between the high and low Intensity bulbs for the 1^st^ graders, p < 0.001, a mean of 2.1 for the 4^th^ graders, p < 0.001, and a mean of 0.3 for the 6^th^ graders, p = 0.5). In addition, there was a significant interaction between Duration and Intensity, *F*(1, 42) = 8.7, *p* = .005, ηp2 = .17, due to a significant difference between the high and low magnitudes in the high intensity light bulb conditions in comparison to a marginally significant difference in the low intensity light bulb conditions (10.8, p = 0.003, vs. 8.9 seconds, p = 0.06, respectively). None of the remaining effects was significant (*p* ≥ .51).

### Discussion

Despite the fact that experiment two was not an exact replication of experiment 2 of the Zakay [12] study, the results of both experiments are similar, as both showed a magnitude effect. As Zakay [12] also reported on the interaction between magnitude and duration, the present results are in line with his findings, since a magnitude effect was more prominent in the longer duration, despite the much shorter duration used in Zakay [12]. Unfortunately, the age variable was not included as an independent variable in the magnitude effect-related analysis, so Zakay [12] did not examine whether the magnitude effect was related to age in the same manner as the present study did. These topics are further discussed in the General Discussion.

## General discussion

The current studies aimed to replicate previous studies in which a magnitude effect was observed in children using a simultaneous duration assessment task and a duration reproduction task. To do so, these tasks were administered to a sample of 45 7-12-year olds in two studies. Both studies revealed a pattern of magnitude effects, i.e., an effect of a non-temporal dimension of the stimuli on the assessment of its temporal dimension.

The study results provide several important conclusions. Firstly, the observed magnitude effect pattern in Study One, which used a simultaneous duration assessment task, replicates and supports earlier research conducted by Levin [4], Levin and Gilat [5]. Furthermore, the replication of the magnitude effect observed in Study Two, which used the same stimuli and sample in a duration reproduction task, further confirms this conclusion. Although the effect sizes were relatively large [25], they were comparable to those observed in other studies investigating the magnitude effect [26–28]. Secondly, the demonstration of the magnitude effect among Arabic-speaking children in both studies suggests that it is not dependent on linguistic factors. This finding supports the results reported by Casasanto, Fotakopoulou [8] and Bottini and Casasanto [7], which also suggest that the magnitude effect is independent of such factors. These results further strengthen the robustness of the magnitude effect among children and increase the external validity of replicated studies.

Another key conclusion of the study relates to the finding that younger children exhibit a greater tendency towards duration perception distortion in both experiments. Specifically, in Study One, younger students were more likely to decide that one bulb had been lit longer, and in Study Two, an interaction between age and the magnitude effect was observed. These results are consistent with previous studies that have reported a negative correlation between time distortion and age, including those conducted by Droit-Volet, Clément [2], Mioni, Stablum [6], Droit-Volet and Hallez [3], Levin [4], and Levin and Gilat [5]. The association of age with time perception distortion found in the current studies can be explained by the claim that time perception depends on developmental factors [29–31]. Since it was argued that the assessment of duration is an effortful process and that it is related to attentional factors [13], and there are reports that attentional resources in children increase with age [32], the current results may be related to the enhanced ability, to utilize an attentional based duration assessment method, e.g. the pacemaker mechanism [15].

In addition to the age-related pattern in time perception distortion, the results of both studies also showed a consistent trend of an increased tendency for the magnitude effect to be demonstrated as the longer evaluation durations. These findings are in line with previous research [26–28, 33, 34], including studies by Rammsayer and Verner [26–28] who reported similar results in all three of their studies. The authors of these studies suggest that the magnitude effect is a context-dependent effect. Notably, previous studies that have also demonstrated a greater tendency for the magnitude effect to occur as a function of duration have typically used shorter durations than those employed in the present study. This interesting pattern may suggest that, similar to the finding that a relative, rather than absolute, difference in magnitude is needed for the effect to occur [17–20], the appearance of the effect may also depend on a relative difference in duration. This factor, which is consistent with the context-dependency explanation discussed earlier, could also account for the reversed magnitude effect observed in study 1 for the first graders in the 20-second condition. Further research would be necessary to investigate the intriguing question *whether a mere relative difference in duration is necessary for the increased tendency for the magnitude effect to be demonstrated in the longer evaluation durations*?.

Levin [4] and Levin and Gilat [5] suggest that the magnitude effect observed in a simultaneous duration assessment task among children may be related to mental development, as children have a tendency to consider magnitudes in task-irrelevant dimensions as task-relevant dimensions, due to an inability to distinguish between these dimensions. However, given that magnitude effects are also prevalent among adults [1], we propose that the temporal pacemaker model [35–39] provides a more suitable explanation. According to the pacemaker model, when there is a need to prospectively assess duration, an internal pacemaker is used to accumulate pulses of biological origin to represent the required interval. The accumulated size can later be used to make time-related decisions regarding this interval or to recreate it by creating a similar duration that corresponds to the same number of accumulated pulses [40]. The magnitude effect can be explained using this model by stating that high magnitude stimuli cause a larger number of pulses to accumulate when presented, as opposed to low magnitude stimuli. These processes result in different accumulated sizes for each type of stimuli, ultimately producing the magnitude effect during the decision or reproduction stages [27, 41–44].

In regards to magnitude effect in a simultaneous duration assessment task, this explanation can be easily applied to multiple pacemakers or a single pacemaker with multiple accumulators, i.e., a differential number of counters is accumulated for each pacemaker or accumulator depending on the stimulus associated with it. For example, when assessing the duration of a large stimulus and a small stimulus, a greater number of counters would be accumulated in relation to the former, while both of them are assessed simultaneously, resulting in a magnitude effect during the decision or the reproduction stages. In spite of the fact that this model provides an elegant and simple explanation, more research is required in order to verify it, since other models for the magnitude effect were suggested [1].

The current studies have a few limitations. First, the studies were conducted only on males for logistical and cultural reasons. However, there is no reason to believe that there are significant magnitude effect-related differences between males and females since no gender-related differences have been reported in any of the previous studies on children (e.g., Levin [4], Zakay [12], Zakay and Block [45]). Second, the order of conditions in study two and the order of studies were fixed across participants, also due to logistical considerations, which may have led to a carry-over effects. A potential limitation in both studies is that the durations used differ from those in the original studies they aimed to replicate. While this difference could be considered a confounding factor, we believe it poses a low risk as the durations used in both studies were longer than the "psychological present" [21–23]. As a result, they were subject to the same duration assessment method as similar prospective duration assessment studies [46]. Another potential limitation of the study is the possible confounding factor of the participants’ ability to perform the simultaneous duration assessment task by comparing the onset and offset times of the lighting durations of the two stimuli. While technically possible, it is important to note that the participants were not informed which light would turn on or off first, or for how long the lights would remain on. Therefore, performing such calculations would have been challenging. Instead, prospectively assessing the stimuli would have been a more straightforward approach, especially considering that each participant only performed the task twice.

We would like to conclude by stating that the present studies successfully replicate the results reported by Levin [4] and Levin and Gilat [5] on the existence of magnitude effects in simultaneous duration tasks. The results further reinforce the idea that the effect is age-dependent, indicating the role of attentional development, and is not influenced by language, culture, or duration assessment methods. Finally, we suggest that the magnitude effects observed in simultaneous duration tasks are consistent with the theoretical framework proposed by the pacemaker model.

## Supporting information

S1 File Study data set. (XLSX)
